# Uptake of new antidiabetic medicines in 11 European countries

**DOI:** 10.1186/s12902-021-00798-3

**Published:** 2021-06-25

**Authors:** Nika Mardetko, Urska Nabergoj Makovec, Igor Locatelli, Andrej Janez, Mitja Kos

**Affiliations:** 1 Department of Social Pharmacy, University of Ljubljana, Faculty of Pharmacy, Askerceva cesta 7, 1000 Ljubljana, Slovenia; 2 Department of Endocrinology, Diabetes and Metabolic Diseases, University Medical Centre Ljubljana, Zaloska Cesta 7, 1000 Ljubljana, Slovenia

**Keywords:** Diabetes Mellitus, Glucagon-Like Peptide 1 receptor agonists, Dipeptidyl-Peptidase 4 inhibitors, Sodium-Glucose Transporter 2 inhibitors, Insulins, Market uptake

## Abstract

**Background:**

Several new antidiabetic medicines (GLP-1 receptor agonists, DPP-4 inhibitors, and SGLT-2 inhibitors) have been approved by the European Medicines Agency since 2006. The aim of this study was to evaluate the uptake of new antidiabetic medicines in European countries over a 10-year period.

**Methods:**

The study used IQVIA quarterly value and volume sales data January 2006–December 2016. The market uptake of new antidiabetic medicines together with intensity of prescribing policy for all antidiabetic medicines were estimated for Austria, Croatia, France, Germany, Hungary, Italy, Poland, Slovenia, Spain, Sweden, and the United Kingdom. The following measures were determined: number of available new active substances, median time to first continuous use, volume market share, and annual therapy cost.

**Results:**

All countries had at least one new antidiabetic medicine in continuous use and an increase in intensity of prescribing policy for all antidiabetic medicines was observed. A tenfold difference in median time to first continuous use (3–30 months) was found. The annual therapy cost in 2016 of new antidiabetic medicines ranged from EUR 363 to EUR 769. Among new antidiabetic medicines, the market share of DPP-4 inhibitors was the highest. Countries with a higher volume market share of incretin-based medicines (Spain, France, Austria, and Germany) in 2011 had a lower increase in intensity of prescribing policy. This kind of correlation was not found in the case of SGLT-2 inhibitors.

**Conclusions:**

This study found important differences and variability in the uptake of new antidiabetic medicines in the included countries.

**Supplementary Information:**

The online version contains supplementary material available at 10.1186/s12902-021-00798-3.

## Background

Diabetes is one of the most challenging health problems in Europe. It is one of the leading causes of death, and its macro- and microvascular complications result in population disability and increased healthcare cost [1]. The prevalence and financial burden of diabetes have increased in European countries and another 10 million patients are expected by 2035. However, diabetes prevalence as well as trends in diabetes prevalence vary significantly between European countries, reflecting differences in management of diabetes as well as its financial burden [2].

Diabetes management consists of lifestyle intervention along with pharmacological therapy and routine blood glucose monitoring [3]. Oral antidiabetics are the most commonly used medicines. Metformin is the first line of treatment and is the most widely prescribed antihyperglycemic medicine. A second-line agent will be added to metformin to achieve individualized glycaemic targets in order to prevent diabetes-related chronic complications. According to the American Diabetes Association (ADA) and the European Association for the Study of Diabetes (EASD) the decision on the second-line agent is based on the risk of comorbidities, risk of hypoglycaemia, body weight, medicine cost, adverse effects, or contraindications [4]. The major classes of old antidiabetic medicines include biguanides, insulin secretagogues (sulfonylureas and glinides), insulin sensitizers (thiazolidinediones), α-glucosidase inhibitors, and insulin. New agents approved by the European Medicines Agency (EMA) are incretin-based therapy (glucagon-like peptide-1 (GLP-1) receptor agonists and dipeptidyl peptidase-4 (DPP-4) inhibitors) and sodium-glucose cotransporter (SGLT-2) inhibitors [4, 5]. The introduction of new agents started with the marketing authorisation of GLP-1 receptor agonist exenatide at the end of 2006 [6].

New treatments for diabetes also come at significantly higher prices [7–9]. For example, an approximately 30-fold difference between metformin and the GLP-1 receptor agonists liraglutide and exenatide was observed in France and Switzerland. Important price differences between metformin and new antidiabetic agents were also reported for the United Kingdom (UK) and Germany [7, 10]. Moreover, prescribed antidiabetic medicines already represent the largest part of costs in diabetes management, followed by the costs of managing diabetes complications [1]. Hence, the introduction and uptake level of new antidiabetic medicines in a particular country could be affected by the healthcare system’s financial capabilities and its priorities. Moreover, differences in country-specific health technology assessment processes supporting payers and decision-makers on the adoption and reimbursement of new medicines could significantly affect patient access to new antidiabetic medicines [11, 12].

## Methods

### Aims

This study evaluates the uptake of new antidiabetic medicines in European countries over a 10-year period.

### Selection of medicines

The ATC codes of the A10 group were used to define all antidiabetic medicines. The products included in the study were categorized into three main groups: new antidiabetics (DPP-4 inhibitors, GLP-1 receptor agonists, SGLT-2 inhibitors), insulins and old antidiabetic medicines (the rest of the antidiabetic medicines). Insulin degludec was considered a new medicine among insulins. In addition, the assumption from 2004 that all medicines used in diabetes need to be authorized by a centralized procedure was taken into account [13, 14]. Therefore, medicines containing new active substances for diabetes treatment that were authorized via a centralized procedure at the EMA between 2006 and 2016 were considered new antidiabetic medicines (see Appendix Table S1 for all medicines included in the study). Fixed combinations for which one of the active substances was a new active substance were assigned to the corresponding group of new medicines.

### Data source

The study was based on the IQVIA quarterly database, January 2006 – December 2016. The IQVIA quarterly value sales data in EUR and quarterly volume sales data expressed in days of treatment (DOTs) for the products from the ATC A10 group were analysed for the purpose of the study. DOTs are estimated based on volume in standard unit measure adjusted to the average (or defined) daily dose.

### Selected countries

The study included a set of 11 different European countries in terms of pharmaceutical market value, population size, geographical location in the EU, and diabetes treatment approach. Consequently, Austria, Croatia, France, Germany, Hungary, Italy, Poland, Slovenia, Spain, Sweden, and the UK were selected. For each country, the data were given either as hospital and retail channels separately or as hospital and retail combined. For eight countries, both channels were given separately, whereas data for Sweden were given combined. In the case of Austria and Hungary, only retail sales data were available and hospital panel data were missing.

### Data analysis

#### Number of new antidiabetic medicines and time to their first continuous use

New antidiabetic medicine was considered to be available in a particular country when its sales were detected in the IQVIA quarterly database. Continuous use was defined as constant 1-year product sales. The time to first continuous use was determined based on the number of products containing one of the new active substances for diabetes treatment available in a particular country. Each country median time to first continuous use was therefore determined using a different number of available products. The time difference was calculated between the new antidiabetic medicine authorisation date and the first medicine continuous use date. The quarter of the year within which the medicine authorisation occurred and the quarter of the first continuous use were considered for the time difference calculation. When a medicine’s continuous use was recognized before marketing authorization, this was considered other practices of patient supply, such as compassionate use. However, in such cases the time to first continuous use was the same as the marketing authorization date (quarter of the year). The median times to first continuous use of new antidiabetic medicines were then compared between the countries.

#### Volume market share and annual therapy cost

Based on the volume sales in DOTs, the market share of new antidiabetics, old antidiabetics and all insulins (including insulin degludec) were defined. Annual therapy cost was calculated separately for all three groups by dividing value sales by volume sales (consumption in DOTs) and then multiplying by the intensity of all antidiabetic medicines prescribing policy (see Eq. 1 for the case of new antidiabetic medicines). The annual therapy cost provide the estimation of cost of the annual therapy per patient in each country.

1 $$\scriptsize Annual\ therapy\ cost \left(new\ medicine\right)=\frac{Annual\ value\ sales \left(new\right)}{Annual\ cons.\ in\ DOTs \left(new\right)}\times\ Intensity\ of\ prescribing\ policy$$

#### Intensity of prescribing policy for all antidiabetic medicines

The intensity of prescribing policy is an index allowing comparison of medicine consumption adjusted for the population at risk [15]. In our study, the volume sales data in DOTs represents annual consumption in DOTs per day in the specified population of diabetes patients (Eq. 2). The annual consumption was calculated for all antidiabetic medicines. The number of diabetes population was calculated by multiplying prevalence of diabetes in each country (in %; data from NCD Risk Factor Collaboration [16] with the country’s total population size (WHO Health Expenditure Database [17].

2 $$\scriptsize Intensity\ of\ antidiabetic\ medicines\ prescribing\ policy\ =\ \frac{Annual\ consumption\ in\ DOTs\ /\ 365\ days }{Number\ of\ diabetes\ patients}$$

The value 1 for intensity of prescribing policy implies that all diabetes patients in the country receive a defined (or average) daily dose of antidiabetic medicine every day within a year, indicating an adequate coverage with medicines. The values above and below 1 would indicate medicines’ abundance or sub-optimal utilization per average diabetic patient, respectively.

### Correlation between the intensity of prescribing policy for all antidiabetic medicines and volume market share of new antidiabetic medicines

Correlation analysis was performed to explain between-country differences in the volume market share of new antidiabetic medicines with regard to the intensity of prescribing policy for all antidiabetic medicines. Pearson correlation coefficient with corresponding statistical test was applied. According to the new antidiabetic medicines marketing authorization and market entry dates, the first correlation analysis was related to incretin-based medicines (DPP-4 inhibitors and GLP-1 receptor agonists) from 2007 to 2011. The second correlation analysis was related to SGLT-2 inhibitors from 2012 to 2016. For this purpose, the ratio of the intensity of prescribing policy for 2011 compared to 2007 and the ratio of the intensity of prescribing policy for 2016 compared to 2012 were calculated. In addition, the volume market share of DPP-4 inhibitors and GLP-1 receptor agonists in 2011 and volume market share of SGLT-2 inhibitors in 2016 were applied.

## Results

### Number of new antidiabetic medicines

**Fig. 1:**
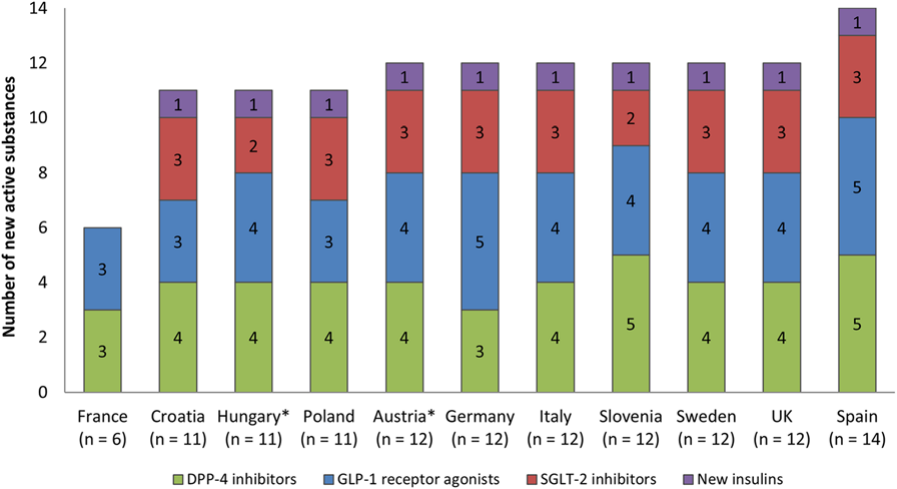
The number of available new antidiabetic active substances in use for each country within the study period. Values in brackets indicate the total number of new active substances. Asterisks indicate countries with only retail sales data available

Fourteen new active substances were introduced from 2006 to 2016; five DPP-4 inhibitors, five GLP-1 receptor agonists, and three SGLT-2 inhibitors. Insulin degludec was considered a new insulin. The availability of new antidiabetic substances in included countries is presented in Fig. 1.

In the set of new antidiabetic medicines there was also a combination of two new active substances, the GLP-1 receptor agonist liraglutide and insulin degludec, which was available in France, Hungary, Germany, Austria, Sweden, and the UK. In further analyses, the combination of liraglutide and degludec was taken into account in the group of GLP-1 analogues.

### Time to first continuous use of new antidiabetic medicines

**Fig. 2:**
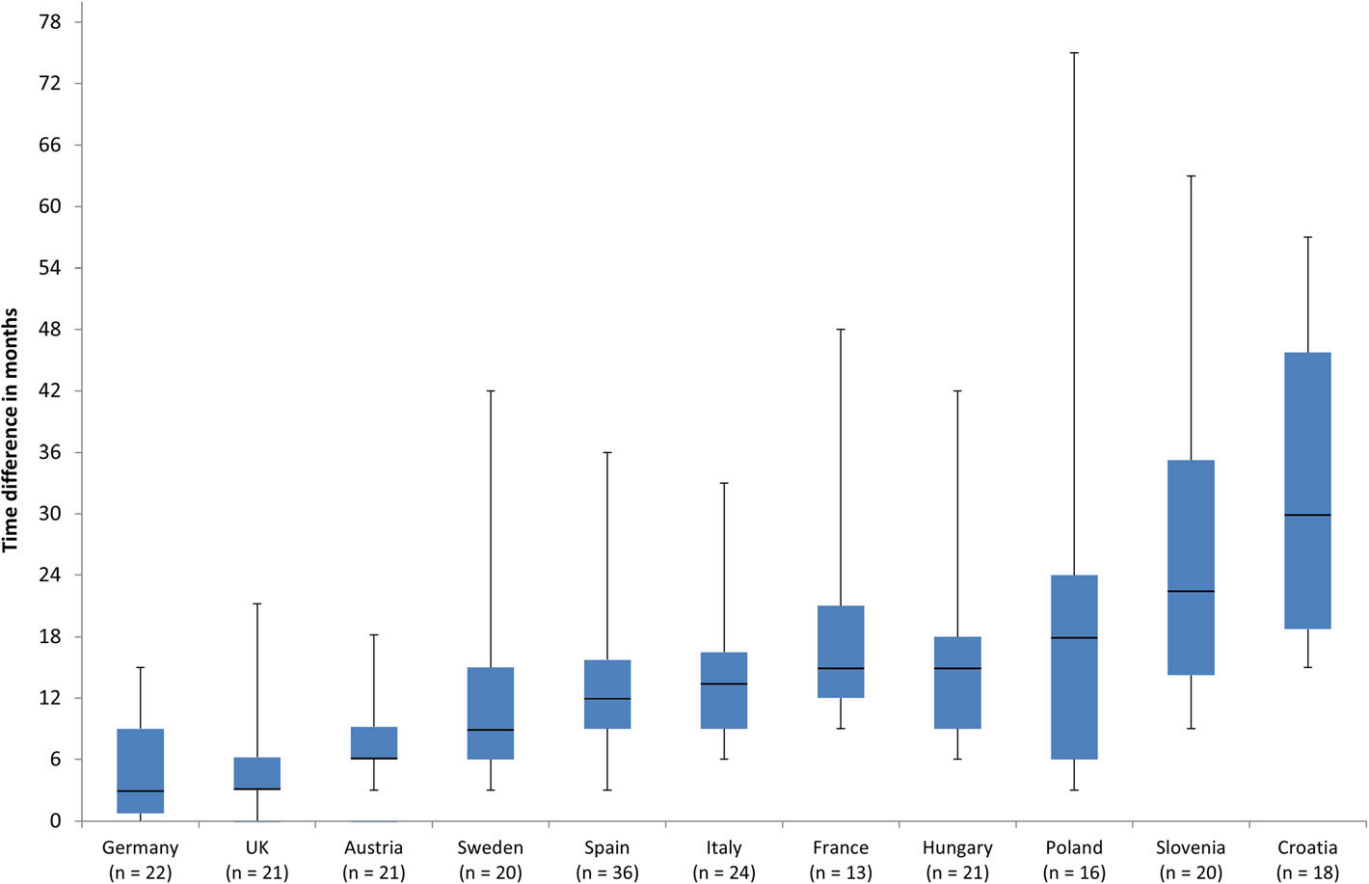
Box plots representing times to first continuous use of new antidiabetic medicines in the included countries. The countries are listed according to increasing median times. Upper and lower bars indicate the values of the third and first quartiles, respectively. The number of products available in each country is given in brackets

The pooled median time to the continuous use of new antidiabetic medicines for all countries together was 13 months. Figure 2 shows the median time to first continuous use of new antidiabetic medicines in each of the selected countries. In this, the estimation of time to first continuous use is based on the country’s available antidiabetics.

A tenfold difference in median time to first continuous use was found among the selected countries. The fastest were Germany and the UK, with a median time of 3 months, followed by Austria and Sweden with a median time of less than 1 year. Apart from the differences in between countries’ median time to first continuous use, a wide range in individual medicine times to first continuous use within a particular country was also found.

### Volume market share and annual therapy cost

The results show a decrease in the volume market share of old antidiabetics, whereas the new antidiabetic medicines’ market share increased. Volume market share of insulins remained more or less unchanged. Among the new antidiabetic medicines, the market share of DPP-4 inhibitors was the highest in all the selected countries. Table 1 shows the volume market share of new and old antidiabetic medicines and all insulins (including insulin degludec), in 2016. The volume market shares were also determined for other study period years and are provided in the Appendix Table S2. Similarly, the proportions of new antidiabetic consumption and expenditure was determined for all years in the study period and are presented in Appendix Figure S1.

**Table 1:** Volume market share and annual therapy cost per patient of new antidiabetic medicines, all insulins, and old antidiabetic medicines in 2016

Country	Volume market share in 2016			Annual therapy cost in 2016		
	**New antidiabetic medicines**^a^** (%)**	**All insulins** **(%)**	**Old antidiabetic medicines (%)**	**New antidiabetic medicines**^a^ **(€)**	**All insulins (€)**	**Old antidiabetic medicines (€)**
**Austria**	26.1 (20.8)^b^	24.1	49.8	460	326	55.8
**Croatia**	10.8 (8.7)^b^	20.8	68.4	371	270	36.9
**France**	17.7 (14.6)^b^	21.1	61.3	480	386	56.6
**Germany**	22.9 (17.5)^b^	36.2	40.9	513	500	32.1
**Hungary**	10.3 (7.5)^b^	26.5	63.2	474	239	45.1
**Italy**	8.8 (6.3)^b^	21.5	69.7	499	326	43.3
**Poland**	1.5 (1.1)^b^	23.5	75.0	363	173	26.9
**Slovenia**	6.6 (4.1)^b^	29.0	63.5	427	284	58.0
**Spain**	26.3 (21.2)^b^	23.4	50.3	456	328	25.7
**Sweden**	9.7 (5.8)^b^	35.2	55.1	769	373	15.9
**UK**	10.6 (6.8)^b^	22.2	67.2	668	390	56.2

Legend: ^a^DPP-4 inhibitors, GLP-1 receptor agonists, and SGLT-2 inhibitors

^b^Values in brackets indicate the volume market share for DPP-4 inhibitors

### Intensity of antidiabetic medicines prescribing policy

**Fig. 3:**
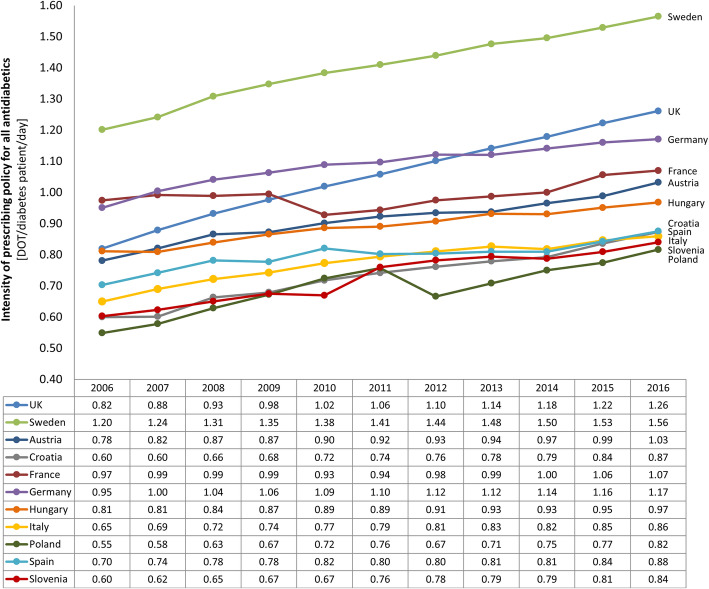
Each country’s intensity of prescribing policy for all antidiabetic medicines in the period 2006–2016. The value 1 for intensity of prescribing policy indicates that all diabetes patients in the country receive a defined (or average) daily dose of antidiabetic medicine every day within a year

Figure 3 shows each country’s intensity of prescribing policy for all antidiabetic medicines. Overall, an increase in intensity of prescribing policy was observed in all the selected countries; the greatest increase was observed in the UK, followed by Poland, Croatia, and Slovenia.

### Correlation between the intensity of prescribing policy for all antidiabetic medicines and volume market share of new antidiabetic medicines

**Fig. 4:**
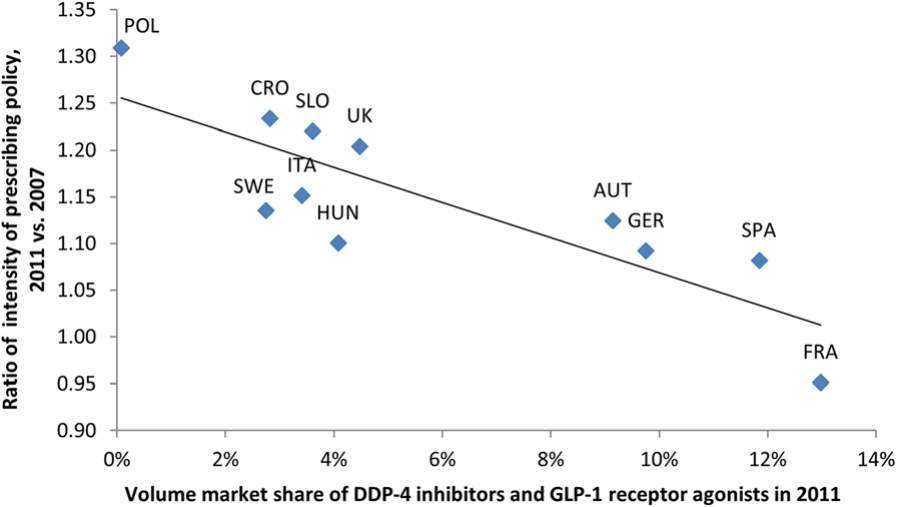
Correlation between the ratio (2011 compared to 2007) of intensity of prescribing policy for all antidiabetic medicines and volume market share of incretin-based medicines (DDP-4 inhibitors and GLP-1 receptor agonists) in 2011

A correlation analysis showed a relatively strong negative and statistically significant correlation (Pearson correlation coefficient = -0.841, *p* = 0.001) between the volume market share of DPP-4 inhibitors and GLP-1 receptor agonists in 2011 and the ratio of intensity of prescribing policy for 2011 compared to 2007. Spain, France, Austria, and Germany had higher volume market shares of DPP-4 inhibitors and GLP-1 receptor agonists, yet a lower ratio of intensity of prescribing policy (Fig. 4.). In contrast, other countries, where volume market share of DPP-4 inhibitors and GLP-1 receptor agonists was lower than 5 %, had a significantly higher ratio of intensity of prescribing policy. However, this kind of correlation was not found in the case of SGLT-2 inhibitors (Pearson correlation coefficient =  -0.351, *p* = 0.290).

## Discussion

This study provides useful insight into differences in intensity of prescribing policy for all antidiabetic medicines and market uptake of new antidiabetic medicines in 11 selected European countries. An increase in the intensity of prescribing policy of antidiabetic medicines was observed in all the countries, suggesting growth in the pharmacological care of diabetic patients and reflects diabetes management as a healthcare priority. It is also in line with the increased number of antidiabetic medicines per patient reported in other recently published literature [18]. However, considering other outcomes of this study, it may also derive from other factors, e.g. changes in country-specific clinical guidelines, national antidiabetic medicines policy, and reimbursement restrictions [12, 14].

At least one new active substance from the group of DPP-4 inhibitors, GLP-1 receptor agonists, and SGLT-2 inhibitors, as well as insulin degludec was in continuous use in all countries included in the study. The only exception was France, where SGLT-2 inhibitors and insulin degludec as sole agent were not available between 2006 and 2016. After our study period, insulin degludec and SGLT-2 inhibitor dapagliflozine were introduced in 2018 and 2020, respectively [19].

Spain, was the only country where all new active substances have been available. In contrast, median times to first continuous use of new antidiabetic medicines differ significantly between countries. As expected, Germany was the fastest in launching new agents, most likely due to the current reimbursement policy of free launch followed by the early benefit assessment [20, 21]. Croatia, Poland, and Slovenia were shown to be the slowest in the introduction and continuous use of new antidiabetic agents. It should be mentioned that all available antidiabetic medicines were considered in this time analysis, which could mean that the first representative of the new antidiabetic group was available soon after marketing authorisation, whereas price negotiations for the subsequent medicines last longer due to payer requirements for the same or even lower price. Additionally, prescribing restrictions could affect the intensity of prescribing policy. For instance, Slovenia introduced SGLT-2 inhibitors with several prescribing restrictions [22], which have been removed in February 2020 and would most probably result in the increase of prescribing.

Although Germany was the fastest in introduction of new antidiabetic agents, it did not have the greatest consumption. It could be linked to the finding, that out of seven evaluated new antidiabetics, only one received an added benefit (non-quantifiable benefit) during the early benefit assessment of the reimbursement procedure by the Federal Joint Committee (G-BA) [23].

The highest volume market shares (around 27 %) of new antidiabetic medicines were observed in Spain and Austria. On the other hand, Poland had the lowest volume market shares, probably due to high patient co-payments for all new antidiabetic medicines [24, 25]. Reserved use of new antidiabetic medicines in almost all of the selected countries could be related to inadequate evidence of benefits according to the price (cost) of new antidiabetic medicines at the investigated time period. The evidence have been usually attributed to surrogate outcomes such as short-term glycaemic control and treatment of adverse effects [20]. Decisions on reimbursement of new antidiabetic agents at that time were therefore based on a lack of evidence, which is less affordable for lower-income countries [26, 27].

Based on the results of correlation analysis (Fig. 4) related to incretin-based medicines (DPP-4 inhibitors and GLP-1 receptor agonists), two groups of countries can be defined. The first group (Spain, Austria, France, and Germany), representing countries with a high volume market share of new antidiabetic medicines (Table 1 and Appendix Table S2) and a slight increase in intensity of prescribing policy from 2007 to 2011 (Fig. 3). The second group consists of all other countries. Therefore, most of the countries evaluated in this study tried to optimize diabetes care through more intense use of old antidiabetic medicines and insulins, and were probably forced to be more conservative in the use of new antidiabetic medicines.

Countries were also shown to differ in the extent of insulin use. Germany, Sweden, and Slovenia, with a volume market share of at least 30 %, are predominant (Table 1 and Table S2 in the Appendix). The literature has already shown that Sweden has a relatively high use of insulin for the treatment of type 2 diabetes compared to other European countries [28]. Up to 38.6 % of the market share of insulins prescribed in primary care practices was also reported in Germany [29]. Insulin treatment is usually started after the oral therapy is already optimized (in double or triple combination and at the maximum tolerated doses) yet fails to achieve optimal glycaemic control. Nonetheless, the insulin initiation is often inappropriately delayed, putting patients to unnecessarily increased risk of complications and potentially reduced quality of life or and life expectancy. This is termed “clinical inertia,” and it can occur due to a number of factors, including clinical concerns (i.e., risk of weight gain, hypoglycaemia, or patient distress), professional concerns (e.g., lack of clinical experience, skills, or confidence in insulin titration), or health system concerns (e.g., competing priorities, regulatory or financial constraints, or a lack of impartial continued medical education) [30, 31].

Furthermore, a great difference in the annual therapy cost of old antidiabetic medicines compared to the annual therapy cost of insulins and new antidiabetic medicines was observed in all the selected countries. The highest annual therapy cost of old antidiabetic medicines was observed in Slovenia, €58. The highest annual therapy cost of new antidiabetic agents was observed in Sweden, €769; however, the annual cost of insulin therapy was significantly lower in Sweden, €373. In contrast, the annual therapy cost of insulins and new antidiabetic agents in Germany were shown to be almost the same, around €500. Nevertheless, the enormous cost gap between the old and new antidiabetic medicines and their financial burden could affect market uptake and consequently patient access to the new agents [8].

### Strengths and limitations

The study included 11 European countries, and address an important therapeutic area with evaluation of all relevant antidiabetic classes in a 10-year study period. It provides useful insight and strengthens the evidence regarding European countries’ variability in introduction and adoption practices of new antidiabetic medicines at the time when limited evidence to assess risk/benefit of new agents were available. Indeed, inclusion of additional countries would contribute to the overall assessment, however the IQVIA data exhibits limitations in terms of the quality and type of data. The database combines two levels of data (retail and hospital consumption), however not in the same manner for all countries. Hence, certain countries were not eligible for inclusion and the study provides estimates of intensity of prescribing policy, and market uptake of new antidiabetic medicines. Furthermore, we were not able to divide the combinations in two different entities, therefore, aiming to detect any new antidiabetic agent available in the selected countries, fixed combinations for which one of the active substances was a new active substance were assigned to the corresponding group of new medicines. Taking a different approach might yield some difference with our current results. Secondly, the study period ended with 2016, when two major clinical trials [32, 33] changed the perspective on GLP-1 receptor agonists and SGLT-2 inhibitors, which resulted in an updated guidelines on diabetes management in 2018 [4]. Extension of the study period would provide additional insights and comparisons into how the new evidence influenced trends in intensity of prescribing policy, volume market shares and annual therapy cost of all antidiabetics classes. However, the availability of data limited the scope of our study.

## Conclusions

All the countries had at least one new active substance among the DPP-4 inhibitors, GLP-1 receptor agonists, SGLT-2 inhibitors, and insulin degludec and overall growth in medication therapy for diabetic patients, shown through the increased intensity of prescribing policy, was observed. Nonetheless, the study found important differences in the uptake of new antidiabetic medicines. A similar comparative study using recent data would introduce new evidence on the evolution and changes of trends detected in this study.

## Supplementary Information

**Additional file 1.**

## Data Availability

Data obtained under license from the following IQVIA information service: IQVIA MIDAS Quarterly Sales Data, January 2006 – December 2016, IQVIA. All Rights Reserved.

## Abbreviations

ATC: Anatomical Therapeutic Chemical (ATC) Classification
DOT: Days of treatment
DPP-4 inhibitors: Dipeptidyl Peptidase-4 inhibitors
EMA: European Medicines Agency
GLP-1 receptor agonists: Glucagon-like Peptide receptor agonists
SGLT-2 inhibitors: Sodium-Glucose transporter 2 inhibitors
UK: United Kingdom
WHO: World Health Organization
