# Supplementary material for: Uptake of new antidiabetic medicines in 11 European countries
Source: BMC Endocr Disord. 2021 Jun 25;21:127. doi: 10.1186/s12902-021-00798-3 (PMC8235847; doi:10.1186/s12902-021-00798-3)

# APPENDIX

Table S1: List of new antidiabetic medicines containing new active substance authorised between 2006 and 2016.

| **Product name** | **Active substance** | **New active substance** | **Authorisation date** | **Categorization of new**  **antidiabetic medicines** |
| --- | --- | --- | --- | --- |
| EFFICIB | sitagliptin/metformin | sitagliptin | 16 Jul 2008 | DPP-4 inhibitors |
| EUCREAS | vildagliptin/metformin | vildagliptin | 14 Nov 2007 | DPP-4 inhibitors |
| ICANDRA | vildagliptin/metformin | vildagliptin | 01 Dec 2008 | DPP-4 inhibitors |
| INCRESYNC | alogliptin/pioglitazone | alogliptin | 19 Sept 2013 | DPP-4 inhibitors |
| JANUMET | sitagliptin/metformin | sitagliptin | 16 Jul 2008 | DPP-4 inhibitors |
| JENTADUETO | linagliptin/metformin | linagliptin | 20 Jul 2012 | DPP-4 inhibitors |
| KOMBIGLYZE IR/XR | saxagliptin/metformin | saxagliptin | 24 Nov 2011 | DPP-4 inhibitors |
| VELMETIA | sitagliptin/metformin | sitagliptin | 16 Jul 2008 | DPP-4 inhibitors |
| VIPDOMET | alogliptin/metformin | alogliptin | 19 Sept 2013 | DPP-4 inhibitors |
| ZOMARIST | vildagliptin/metformin | vildagliptin | 01 Dec 2008 | DPP-4 inhibitors |
| GALVUS | vildagliptin | vildagliptin | 26 Sept 2007 | DPP-4 inhibitors |
| JALRA | vildagliptin | vildagliptin | 19 Nov 2008 | DPP-4 inhibitors |
| JANUVIA | sitagliptin | sitagliptin | 21 Mar 2007 | DPP-4 inhibitors |
| ONGLYZA | saxagliptin | saxagliptin | 01 Oct 2009 | DPP-4 inhibitors |
| RISTABEN | sitagliptin | sitagliptin | 15 Mar 2010 | DPP-4 inhibitors |
| RISTFOR | sitagliptin/metformin | sitagliptin | 15 Mar 2010 | DPP-4 inhibitors |
| TESAVEL | sitagliptin | sitagliptin | 10 Jan 2008 | DPP-4 inhibitors |
| TRADJENTA | linagliptin | linagliptin | 24 Aug 2011 | DPP-4 inhibitors |
| VIPIDIA | alogliptin benzoate | alogliptin | 19 Sept 2013 | DPP-4 inhibitors |
| XELEVIA | sitagliptin | sitagliptin | 21 Mar 2007 | DPP-4 inhibitors |
| XILIARX | vildagliptin | vildagliptin | 19 Nov 2008 | DPP-4 inhibitors |
| XULTOPHY | insulin degludec / liraglutide | insulin degludec / liraglutide | 18 Sept 2014 | GLP-1 analogues |
| BYDUREON | exenatide | exenatide | 17 Jun 2011 | GLP-1 analogues |
| BYETTA | exenatide | exenatide | 20 Nov 2006 | GLP-1 analogues |
| LYXUMIA | lixisenatide | lixisenatide | 01 Feb 2013 | GLP-1 analogues |
| TANZEUM | albiglutide | albiglutide | 21 Mar 2014 | GLP-1 analogues |
| TRULICITY | dulaglutide | dulaglutide | 21 Nov 2014 | GLP-1 analogues |
| VICTOZA | liraglutid | liraglutid | 30 Jun 2009 | GLP-1 analogues |
| RYZODEG | insulin degludec / insulin aspart | insulin degludec / insulin aspart | 21 Jan 2013 | other new medicines |
| TRESIBA | insulin degludec | insulin degludec | 21 Jan 2013 | other new medicines |
| EBYMECT | dapagliflozin/metformin | dapagliflozin | 16 Nov 2015 | SGLT-2 inhibitors |
| SYNJARDY | empagliflozin/metformin | empagliflozin | 27 May 2015 | SGLT-2 inhibitors |
| VOKANAMET | canagliflozin/metformin | canagliflozin | 23 Apr 2014 | SGLT-2 inhibitors |
| XIGDUO IR/XR | dapagliflozin/metformin | dapagliflozin | 16 Jan 2014 | SGLT-2 inhibitors |
| EDISTRIDE | dapagliflozin | dapagliflozin | 09 Nov 2015 | SGLT-2 inhibitors |
| FORXIGA | dapagliflozin | dapagliflozin | 12 Nov 2012 | SGLT-2 inhibitors |
| INVOKANA | canagliflozin | canagliflozin | 15 Nov 2013 | SGLT-2 inhibitors |
| JARDIANCE | empagliflozin | empagliflozin | 22 May 2014 | SGLT-2 inhibitors |
| QTERN | saxagliptin/dapagliflozin | saxagliptin/dapagliflozin | 15 Jul 2016 | SGLT2 inhibitors and DPP-4 inhibitors |
| GLYXAMBI | empagliflozin/linagliptin | empagliflozin/linagliptin | 11 Nov 2016 | SGLT2 inhibitors and DPP-4 inhibitors |

Table S2. Volume market share of old and new antidiabetic agents and insulins in a period from 2006 to 2016.

| **Country** | **Year** | **All insulins** | **Old antidiabetic medicines** | **New antidiabetic medicines** | **DDP-4 inhibitors** | **GLP-1 receptor agonists** | **SGLT-2 inhibitors** |
| --- | --- | --- | --- | --- | --- | --- | --- |
| **AUSTRIA** | 2006 | 26.1% | 73.9% | 0.0% |  |  |  |
|  | 2007 | 25.6% | 74.4% | 0.0% | 0.04% | 0.002% |  |
|  | 2008 | 25.3% | 73.6% | 1.0% | 1.0% | 0.02% |  |
|  | 2009 | 25.7% | 71.2% | 3.1% | 3.0% | 0.03% |  |
|  | 2010 | 25.5% | 69.0% | 5.5% | 5.4% | 0.05% |  |
|  | 2011 | 25.0% | 65.8% | 9.1% | 9.0% | 0.1% |  |
|  | 2012 | 24.7% | 63.0% | 12.3% | 12.0% | 0.2% |  |
|  | 2013 | 24.6% | 59.5% | 15.9% | 15.4% | 0.5% | 0.1% |
|  | 2014 | 24.5% | 56.3% | 19.1% | 17.9% | 0.6% | 0.7% |
|  | 2015 | 24.6% | 53.4% | 22.1% | 19.6% | 0.8% | 1.7% |
|  | 2016 | 24.1% | 49.8% | 26.1% | 20.8% | 1.0% | 4.3% |
| **CROATIA** | 2006 | 45.7% | 54.3% | 0.0% |  |  |  |
|  | 2007 | 24.0% | 76.0% | 0.0% |  |  |  |
|  | 2008 | 23.8% | 76.2% | 0.0% |  |  |  |
|  | 2009 | 22.9% | 77.0% | 0.1% | 0.1% |  |  |
|  | 2010 | 22.9% | 76.0% | 1.1% | 1.1% | 0.003% |  |
|  | 2011 | 22.3% | 74.9% | 2.8% | 2.7% | 0.1% |  |
|  | 2012 | 22.1% | 73.9% | 4.0% | 3.8% | 0.2% |  |
|  | 2013 | 21.6% | 72.6% | 5.8% | 5.5% | 0.2% |  |
|  | 2014 | 21.3% | 71.1% | 7.6% | 7.1% | 0.5% | 0.02% |
|  | 2015 | 20.9% | 69.5% | 9.7% | 8.5% | 0.9% | 0.3% |
|  | 2016 | 20.8% | 68.4% | 10.8% | 8.7% | 1.2% | 0.8% |
| **FRANCE** | 2006 | 16.8% | 83.2% | 0.0% |  |  |  |
|  | 2007 | 17.4% | 82.6% | 0.0% |  |  |  |
|  | 2008 | 17.9% | 81.3% | 0.8% | 0.7% | 0.1% |  |
|  | 2009 | 17.9% | 78.8% | 3.3% | 2.8% | 0.5% |  |
|  | 2010 | 19.7% | 71.9% | 8.4% | 7.4% | 1.0% |  |
|  | 2011 | 19.8% | 67.2% | 13.0% | 11.4% | 1.6% |  |
|  | 2012 | 20.0% | 64.8% | 15.3% | 13.3% | 2.0% |  |
|  | 2013 | 20.4% | 63.5% | 16.1% | 13.9% | 2.2% |  |
|  | 2014 | 20.6% | 62.9% | 16.4% | 14.1% | 2.4% |  |
|  | 2015 | 20.9% | 62.1% | 17.0% | 14.4% | 2.6% |  |
|  | 2016 | 21.1% | 61.3% | 17.7% | 14.6% | 3.1% |  |
| **GERMANY** | 2006 | 42.1% | 57.9% |  |  |  |  |
|  | 2007 | 41.3% | 58.4% | 0.4% | 0.3% | 0.1% |  |
|  | 2008 | 40.3% | 58.0% | 1.6% | 1.4% | 0.3% |  |
|  | 2009 | 39.4% | 57.2% | 3.5% | 3.0% | 0.4% |  |
|  | 2010 | 38.4% | 55.2% | 6.4% | 5.6% | 0.8% |  |
|  | 2011 | 38.1% | 52.1% | 9.8% | 8.8% | 0.9% |  |
|  | 2012 | 37.9% | 49.6% | 12.4% | 11.2% | 1.2% | 0.001% |
|  | 2013 | 38.1% | 47.1% | 14.8% | 13.1% | 1.3% | 0.4% |
|  | 2014 | 38.2% | 44.6% | 17.2% | 14.9% | 1.3% | 1.0% |
|  | 2015 | 37.4% | 42.6% | 20.0% | 16.4% | 1.6% | 2.0% |
|  | 2016 | 36.2% | 40.9% | 22.9% | 17.5% | 2.0% | 3.4% |
| **HUNGARY** | 2006 | 23.4% | 76.6% |  |  |  |  |
|  | 2007 | 23.2% | 76.8% |  |  |  |  |
|  | 2008 | 24.0% | 75.9% | 0.1% | 0.1% | 0.0% | 0.0% |
|  | 2009 | 24.6% | 74.5% | 1.0% | 1.0% | 0.0% | 0.0% |
|  | 2010 | 25.5% | 72.2% | 2.3% | 2.2% | 0.1% | 0.0% |
|  | 2011 | 26.1% | 69.8% | 4.1% | 3.9% | 0.2% | 0.0% |
|  | 2012 | 26.7% | 68.3% | 5.0% | 4.7% | 0.3% | 0.0% |
|  | 2013 | 26.5% | 67.5% | 6.0% | 5.6% | 0.4% | 0.0% |
|  | 2014 | 26.7% | 66.1% | 7.2% | 6.5% | 0.7% | 0.0% |
|  | 2015 | 27.0% | 64.6% | 8.5% | 7.0% | 0.9% | 0.5% |
|  | 2016 | 26.5% | 63.2% | 10.3% | 7.5% | 1.2% | 1.6% |
| **ITALY** | 2006 | 22.8% | 77.2% |  |  |  |  |
|  | 2007 | 23.5% | 76.5% |  |  |  |  |
|  | 2008 | 23.2% | 76.5% | 0.3% | 0.2% | 0.1% |  |
|  | 2009 | 23.1% | 76.0% | 0.9% | 0.6% | 0.2% |  |
|  | 2010 | 22.8% | 75.7% | 1.5% | 1.2% | 0.3% |  |
|  | 2011 | 22.3% | 74.3% | 3.4% | 2.7% | 0.7% |  |
|  | 2012 | 22.1% | 72.7% | 5.2% | 4.2% | 1.0% |  |
|  | 2013 | 21.8% | 71.6% | 6.5% | 5.4% | 1.2% |  |
|  | 2014 | 21.8% | 71.8% | 6.4% | 5.3% | 1.1% |  |
|  | 2015 | 21.9% | 70.9% | 7.3% | 5.8% | 1.2% | 0.2% |
|  | 2016 | 21.5% | 69.7% | 8.8% | 6.3% | 1.4% | 1.1% |
| **POLAND** | 2006 | 30.8% | 69.2% |  |  |  |  |
|  | 2007 | 30.5% | 69.5% |  |  |  |  |
|  | 2008 | 29.2% | 70.8% | 0.005% | 0.005% |  |  |
|  | 2009 | 28.6% | 71.4% | 0.02% | 0.02% | 0.001% |  |
|  | 2010 | 28.0% | 71.9% | 0.04% | 0.04% | 0.004% |  |
|  | 2011 | 28.4% | 71.5% | 0.1% | 0.06% | 0.01% |  |
|  | 2012 | 25.1% | 74.8% | 0.1% | 0.11% | 0.02% |  |
|  | 2013 | 26.0% | 73.8% | 0.2% | 0.20% | 0.02% | 0.02% |
|  | 2014 | 25.5% | 74.1% | 0.4% | 0.33% | 0.02% | 0.05% |
|  | 2015 | 24.7% | 74.5% | 0.8% | 0.66% | 0.03% | 0.1% |
|  | 2016 | 23.5% | 75.0% | 1.5% | 1.10% | 0.06% | 0.4% |
| **SPAIN** | 2006 | 24.2% | 75.8% |  |  |  |  |
|  | 2007 | 24.2% | 75.8% | 0.0007% | 0.0007% |  |  |
|  | 2008 | 23.6% | 75.6% | 0.8% | 0.8% | 0.002% |  |
|  | 2009 | 23.6% | 72.5% | 3.9% | 3.8% | 0.1% |  |
|  | 2010 | 23.6% | 68.3% | 8.1% | 7.8% | 0.3% |  |
|  | 2011 | 23.8% | 64.4% | 11.8% | 11.5% | 0.3% |  |
|  | 2012 | 24.2% | 61.0% | 14.8% | 14.1% | 0.7% |  |
|  | 2013 | 24.7% | 57.6% | 17.7% | 16.7% | 0.9% | 0.001% |
|  | 2014 | 24.3% | 55.7% | 20.0% | 18.5% | 1.3% | 0.2% |
|  | 2015 | 24.3% | 53.2% | 22.5% | 19.8% | 1.6% | 1.2% |
|  | 2016 | 23.4% | 50.3% | 26.3% | 21.2% | 1.8% | 3.3% |
| **SLOVENIA** | 2006 | 32.5% | 67.5% | 0.0% |  |  |  |
|  | 2007 | 33.3% | 66.7% | 0.0% |  |  |  |
|  | 2008 | 34.0% | 65.9% | 0.1% | 0.1% |  |  |
|  | 2009 | 34.3% | 64.7% | 0.9% | 0.9% | 0.0% |  |
|  | 2010 | 34.0% | 64.2% | 1.8% | 1.6% | 0.3% |  |
|  | 2011 | 31.6% | 64.8% | 3.6% | 3.2% | 0.4% |  |
|  | 2012 | 31.0% | 64.3% | 4.8% | 4.2% | 0.6% |  |
|  | 2013 | 30.0% | 65.2% | 4.8% | 4.1% | 0.7% |  |
|  | 2014 | 30.3% | 65.2% | 4.5% | 3.7% | 0.8% | 0.1% |
|  | 2015 | 30.4% | 64.1% | 5.4% | 4.0% | 1.0% | 0.4% |
|  | 2016 | 29.9% | 63.5% | 6.6% | 4.1% | 1.2% | 1.3% |
| **SWEDEN** | 2006 | 40.5% | 59.5% |  |  |  |  |
|  | 2007 | 40.4% | 59.6% | 0.1% | 0.08% | 0.01% |  |
|  | 2008 | 39.9% | 59.4% | 0.7% | 0.60% | 0.07% |  |
|  | 2009 | 39.5% | 59.3% | 1.2% | 1.04% | 0.12% |  |
|  | 2010 | 39.3% | 59.0% | 1.8% | 1.49% | 0.27% |  |
|  | 2011 | 38.5% | 58.8% | 2.7% | 2.05% | 0.70% |  |
|  | 2012 | 38.1% | 58.3% | 3.6% | 2.49% | 1.13% |  |
|  | 2013 | 37.7% | 57.7% | 4.6% | 3.07% | 1.46% | 0.03% |
|  | 2014 | 37.6% | 56.6% | 5.8% | 3.77% | 1.71% | 0.29% |
|  | 2015 | 36.6% | 55.9% | 7.5% | 4.70% | 2.12% | 0.70% |
|  | 2016 | 35.2% | 55.1% | 9.7% | 5.77% | 2.67% | 1.30% |
| **UK** | 2006 | 30.0% | 70.0% |  |  |  |  |
|  | 2007 | 29.6% | 70.4% | 0.1% | 0.04% | 0.02% |  |
|  | 2008 | 28.7% | 70.8% | 0.5% | 0.3% | 0.2% |  |
|  | 2009 | 27.6% | 70.9% | 1.5% | 1.0% | 0.5% |  |
|  | 2010 | 26.4% | 70.5% | 3.1% | 2.1% | 1.0% |  |
|  | 2011 | 24.5% | 71.0% | 4.5% | 3.2% | 1.3% |  |
|  | 2012 | 23.8% | 70.6% | 5.7% | 4.2% | 1.5% | 0.0001% |
|  | 2013 | 23.2% | 70.1% | 6.7% | 5.0% | 1.6% | 0.1% |
|  | 2014 | 23.0% | 69.2% | 7.8% | 5.8% | 1.6% | 0.4% |
|  | 2015 | 22.7% | 68.1% | 9.2% | 6.4% | 1.6% | 1.2% |
|  | 2016 | 22.2% | 67.2% | 10.6% | 6.8% | 1.7% | 2.1% |


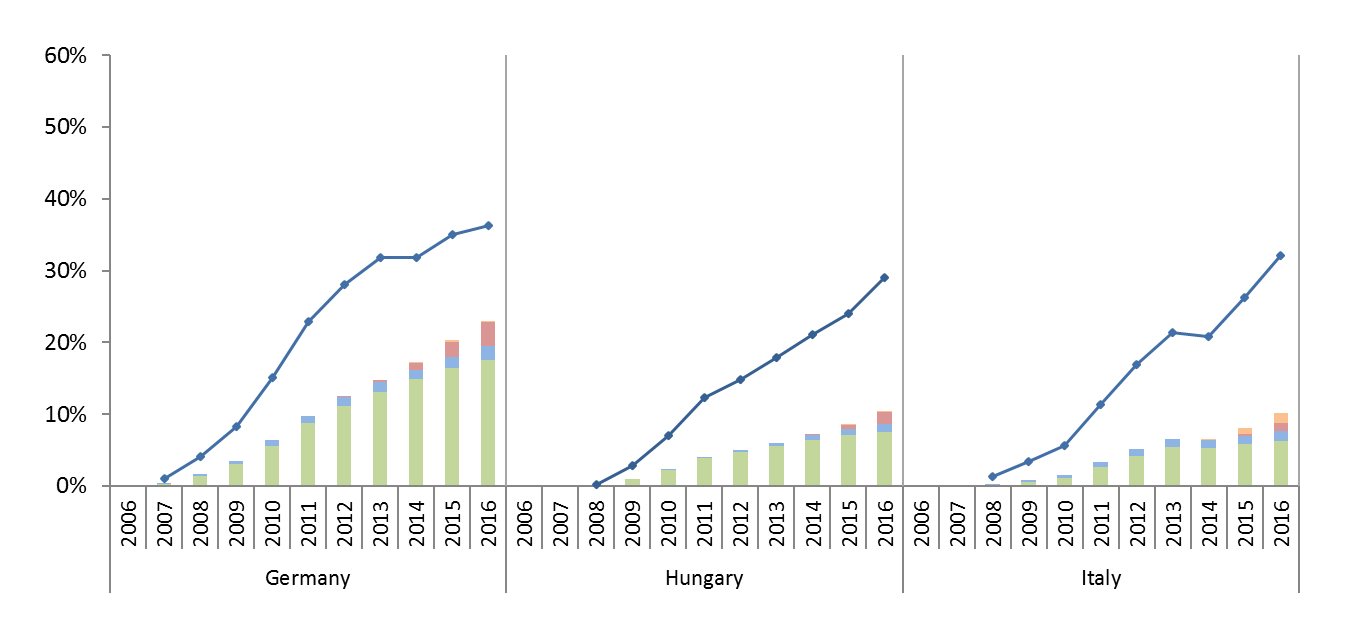

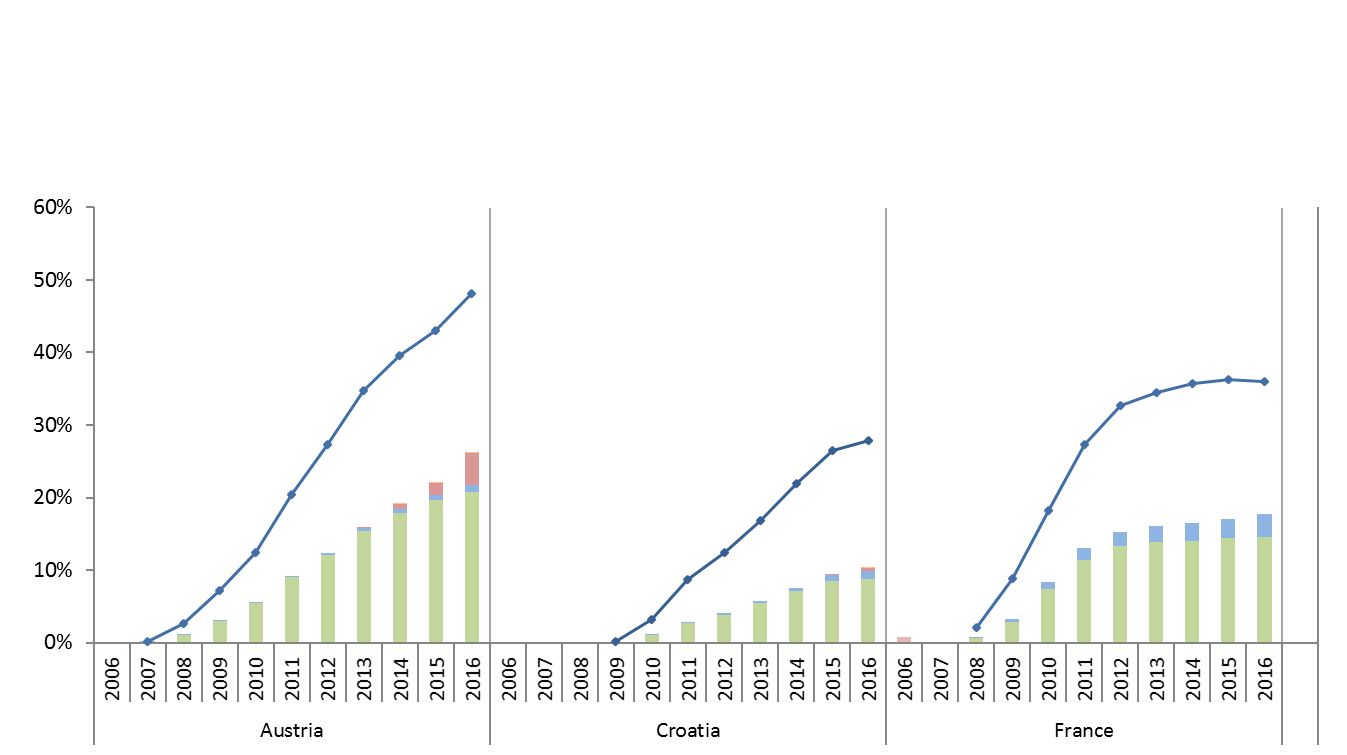

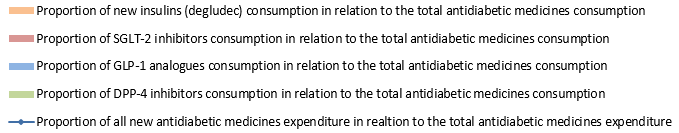
Fi**gure S1: Trend of the proportion of new antidiabetic medicines consumption (bars) and trend of the proportion of new antidiabetic medicines expenditure in relation to the total antidiabetic medicines expenditure (line) in every included country for the period 2006-2016.**


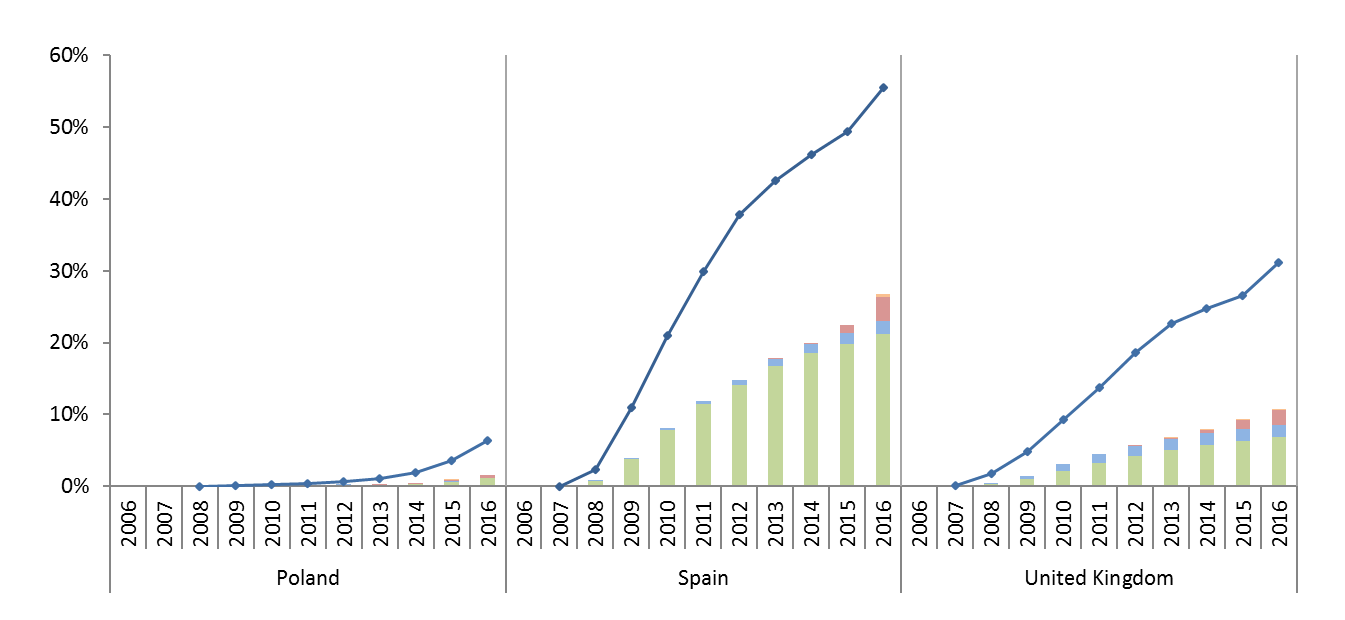

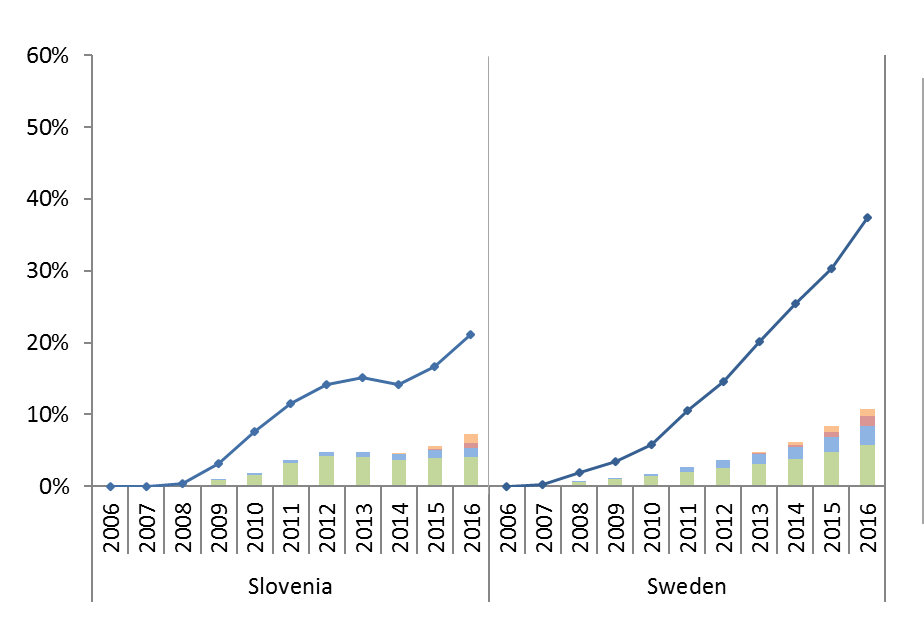

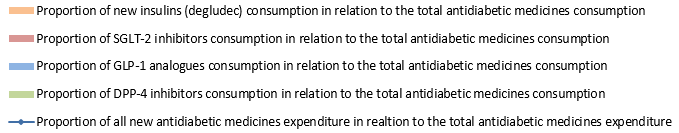

Supplement: Supplementary file 1 — Additional file 1. [file 12902_2021_798_MOESM1_ESM.docx]
